# Neuron-Specific IMP2 Overexpression by Synapsin Promoter-Driven AAV9: A Tool to Study Its Role in Axon Regeneration

**DOI:** 10.3390/cells10102654

**Published:** 2021-10-05

**Authors:** Sarah Blizard, Danielle Park, Natalie O’Toole, Sheeva Norooz, Martin Dela Torre, Young Son, Adam Holstein, Scarlett Austin, Joshua Harman, Samantha Haraszti, Daved Fared, Mei Xu

**Affiliations:** Department of Bio-Medical Sciences, Philadelphia College of Osteopathic Medicine, 4170 City Avenue, Philadelphia, PA 19131, USA; blizardsb1@gmail.com (S.B.); daniseopark@gmail.com (D.P.); natalieot@pcom.edu (N.O.); sheevano@pcom.edu (S.N.); martinde@pcom.edu (M.D.T.); youngso@pcom.edu (Y.S.); adamho@pcom.edu (A.H.); scarlettau@pcom.edu (S.A.); joshuahar@pcom.edu (J.H.); samanthaha@pcom.edu (S.H.); df7366@pcom.edu (D.F.)

**Keywords:** adeno-associated viral vector, synapsin promoter, axon regeneration, Insulin-like growth factor II mRNA-binding protein 2, β-actin mRNA localization

## Abstract

Insulin-like growth factor II mRNA-binding protein (IMP) 2 is one of the three homologues (IMP1-3) that belong to a conserved family of mRNA-binding proteins. Its alternative splice product is aberrantly expressed in human hepatocellular carcinoma, and it is therefore identified as HCC. Previous works have indicated that IMP1/ZBP1 (zipcode binding protein) is critical in axon guidance and regeneration by regulating localization and translation of specific mRNAs. However, the role of IMP2 in the nervous system is largely unknown. We used the synapsin promoter-driven adeno-associated viral (AAV) 9 constructs for transgene expression both in vitro and in vivo. These viral vectors have proven to be effective to transduce the neuron-specific overexpression of IMP2 and HCC. Applying this viral vector in the injury-conditioned dorsal root ganglion (DRG) culture demonstrates that overexpression of IMP2 significantly inhibits axons regenerating from the neurons, whereas overexpression of HCC barely interrupts the process. Quantitative analysis of binding affinities of IMPs to β-actin mRNA reveals that it is closely associated with their roles in axon regeneration. Although IMPs share significant structural homology, the distinctive functions imply their different ability to localize specific mRNAs and to regulate the axonal translation.

## 1. Introduction

Insulin-like growth factor II mRNA-binding proteins (IMPs) including isoforms IMP1-3 consist of two conical RNA-recognition motifs and four K-homology (KH) RNA binding domains, which are key elements for RNA binding [1,2]. IMP1 is also known as the zipcode binding protein (ZBP1) [1], whereas an alternative splice product of IMP2 is aberrantly expressed in human hepatocellular carcinoma and therefore identified as HCC or auto-antigen p62 [3]. IMPs play an essential role in embryonic growth and development. IMP1 and IMP3 are abundant in the embryonic brain, spinal cord, and dorsal root ganglion (DRG), as well as in other organs, and drop to barely detectable levels towards the end of embryogenesis [4,5,6]. IMP2 expression is similar to that of IMP1 and IMP3 during embryogenesis, but it is sustained in the neural tissues throughout life [5,7,8]. ZBP1 (IMP1) binds to β-actin mRNA via the 3′ UTR and localizes it in the distal cellular compartment, such as nerve axons, while repressing its translation [9,10]. Our previous study demonstrated that axon regeneration capacity was compromised in the adult DRG neurons and crushed sciatic nerve of heterozygous *ZBP1* (*IMP1*) mice due to reduced axonal ß-actin mRNA localization and its local translation, suggesting a role of ZBP1/IMP1 in facilitating axon regrowth [6]. With structural similarity to its isoforms, the role of persistent expression of IMP2 in the adult neural tissue is, however, yet to be known. Several studies indicate IMP2 in the regulation of neural precursor cell differentiation of the neocortex and guidance of axon pathway-finding during development [8,11]. Other studies provide evidence that links IMP2 to etiology of type 2 diabetes. For example, IMP2 knockout mice exhibit insulin sensitivity and resistance to diet-induced obesity [12,13,14,15]. Additionally, overexpression of IMP2 has been associated with cancer cell proliferation and migration, and its role in poor prognosis of cancers has been suggested [16,17,18].

Injury to axons triggers an array of cellular and molecular responses that involve both growth promoting and inhibiting proteins. Some of these proteins are derived from translation of localized mRNAs in the axons. In the injury-conditioned DRG neuron culture, over 200 localized mRNAs and locally translated proteins in the regenerating axons are identified, which include cytoskeletal molecules, such as β-actin, peripherin, and tropmomyosin [19,20,21]. The robust axonal mRNA localization and local protein synthesis fulfill several functions. Injury-induced local protein synthesis contributes to growth cone formation and allows the distal process to communicate with the cell body (e.g., importin β1 and RanBP1), and locally synthesized mTOR controls axonal mRNA translation [22,23,24,25,26]. In fact, local mRNA translation occurs in both developing and mature axons. The developing axons present a translatome of axon-specific mRNAs, whereas the mature axons comprise a complex translatome related to axonal survival, neurotransmission, and neurodegenerative diseases in the retinal ganglion cells [27].

Adeno-associated virus (AAV) is one of the most commonly used vectors for gene delivery [28]. AAV can transduce non-dividing cells such as neurons with high efficiency and long-lasting expression [29,30]. AAV serotypes exhibit different tissue tropism [31]. Out of all the serotypes, AAV9 has been extensively utilized in delivering genes to the neural tissue [32,33]. It has been shown that intravenous injection of AAV9 can transduce the neurons and astrocytes in the spinal cord, DRG, and brain [34]. Recently, significant attention has been given to its application to treat neurodegenerative diseases. Spinal subpial injection of AAV9 is adapted in gene silencing treatment in the mutant *SOD1* mouse model of familial amyotrophic lateral sclerosis [35]. AAV9 gene therapy for giant axonal neuropathy is currently in clinical trial [36]. We previously tested several other approaches for transgene expression, such as using nucleofection in the DRG neuron culture and rabies-G pseudotyped lentiviral vector in the severed sciatic nerve. We noticed their neurotoxicity and non-neuronal expression. Taking advantage of a neuron-specific promoter synapsin, we applied the synapsin promoter-driven AAV9 constructs both in vitro and in vivo [32,37]. These viral vectors can effectively transduce the neuron-specific transgene expression. Our data show that IMPs have significantly different binding affinity to β-actin mRNA, although they share homologous RNA binding domains. Unlike IMP1 and HCC, IMP2 plays an inhibitory role in the regenerative process of mature axons, which could be attributed to their mRNA target preferences.

## 2. Materials and Methods

### 2.1. The Sciatic Nerve Crush, Injury-Conditioned DRG Culture and Viral Transduction

In anesthetized adult C57/BL6 mice, the skin of right midthighs were incised, the muscles were retracted, and the sciatic nerves were exposed. The right sciatic nerves were crushed with an un-serrated hemostatic forceps for 30 sec, and then the incisions were carefully closed. The left sciatic nerves were exposed but not crushed and were used as a sham control. Some mice received 5 µL of 8.5 × 10^12^ GC/mL of AAV9.hSyn.YFP injection into the crush site during the surgery. The mice were then euthanized and perfused transcardially with phosphate-buffered saline (PBS) followed by 4% paraformaldehyde 5–7 days after surgery. The sciatic nerves and DRGs were harvested and processed for either cryosectioning at 20 µm or used directly for whole mount immunofluorescence. Other mice were euthanized by inhalation of overdose CO_2_ 5–7 days after surgery. The sciatic nerves were harvested for β-actin mRNA quantification. L4–L6 DRGs were dissected and dissociated. The DRG neurons were plated on polylysine/laminin-coated coverslips in DME/F12 medium containing N1 supplement and 10% horse serum and cultured at 37 °C in a humidified 5% CO_2_ incubator. Replication deficient constructs AAV9.hSyn.YFP, AAV9.hSyn.YFP-IMP2, and AAV9.hSyn.YFP-HCC were produced by Penn Vector Core, University of Pennsylvania Perelman School of Medicine, Philadelphia, PA, USA. They were applied to the culture at MOI of 5 × 10^4^ at 1 day in vitro (DIV) and incubated for 3 days.

All procedures involving animals were conducted according to the guidelines set forth by U.S.A. Institutional Animal Care and Use Committee (IACUC) Guidebook and approved by the IACUC of the Philadelphia College of Osteopathic Medicine.

### 2.2. Immunofluorescence

The whole-mount sciatic nerves were washed in 0.3% Triton X-100/PBS before blocked in 10% bovine serum albumin (BSA) overnight. They were incubated in mouse anti-GAP43 antibody (1:200, Thermo Fisher Scientific, Waltham, MA, USA) for 48 h at 4 °C. After a stringent wash, anti-mouse IgG (H + L) Alexa Fluor^®^ 594 (1:500, Jackson ImmunoResearch, West Grove, PA, USA) was applied to the nerves and incubated in a dark box for 24 h at 4 °C. The nerves were then washed, mounted on glass slides in Vectashield^®^ (Vector Laboratories, Burlingame, CA, USA), and viewed with an Olympus Fluoview 1000 confocal microscope.

The DRG neurons on coverslips were rinsed briefly with PBS and fixed in 4% paraformaldehyde/PBS for 20 min at room temperature. They were then rinsed and blocked in 2% BSA. Mouse anti-tau antibody (1:500, Sigma-Aldrich, St. Louis, MO, USA) was applied to the coverslips and incubated in a humid chamber for 2 h, followed by anti-mouse IgG (H + L) Alexa Fluor^®^ 594 (1:1000, Jackson ImmunoResearch, West Grove, PA, USA) for 45 min. They were then rinsed and stained for DAPI before being coverslipped for imaging.

### 2.3. Data Analysis

The coverslips with the DRG neurons were viewed under a high-resolution inverted microscope. The DRG neurons transduced to express YFP by AAV9.hSyn.YFP, AAV9.hSyn.YFP-IMP2 and AAV9.hSyn.YFP-HCC were individually identified, and the images were captured. Their axons immunostained for tau were traced and quantified with ImageJ software. The data collection and blind analyses were conducted by four independent researchers. Statistical analysis was performed using one-way ANOVA with Tukey’s HSD post hoc test.

### 2.4. N2A Cell Culture and DNA Transfection

N2A cells were cultured in DMEM medium (Thermo Fisher Scientific, Waltham, MA, USA) supplemented with 10% FBS and 100 U/mL penicillin–streptomycin. After the cells reached 80% confluence, the medium was removed and replaced with Opti-MEM^®^ (Gibco, Gaithersburg, MD, USA). The plasmids of pGFP-C1, pC1-GFP-IMP1, pC1-YFP-IMP2, pC1-YFP-HCC, and pC1-GFP-IMP3 were transfected with Lipofectamine^®^2000 according to the manufacturer’s instruction (Thermo Fisher Scientific, Waltham, MA, USA). Briefly, 4 µg of each plasmid DNA was diluted in 250 µL of Opti-MEM^®^, while 10 µL of Lipofectamine^®^2000 was diluted in 250µL of Opti-MEM^®^ and incubated for 5 min. The diluted plasmid DNA and Lipofectamine^®^2000 were combined and incubated for 20 min. The DNA–Lipofectamine^®^2000 complexes were then added to the cells and incubated at 37 °C in the incubator for 2 DIV before the cells were harvested.

### 2.5. Western Blot Analysis

Equal amount of protein from the cell lysates was resolved in a 10% polyacrylamide gel and transferred to PVDF membrane. After blocking, the membrane was probed with mouse anti-GFP (1:1000, Thermo Fisher Scientific, Waltham, MA, USA) at 4 °C overnight, followed by horseradish peroxidase-conjugated anti-mouse IgG (H + L) (1:10,000, Bio-Rad, Hercules, CA, USA) for 1 h. The immunoreactivity was then detected with enhanced chemiluminescence reagents (Thermo Fisher Scientific, Waltham, MA, USA) and visualized by ChemiDoc MP (Bio-Rad, Hercules, CA, USA).

### 2.6. Immunoprecipitation and Quantitative Measurement of β-actin mRNA

The cell lysates were pre-cleared. They were then incubated with protein G sepharose beads pre-treated with 4 µg of either mouse monoclonal anti-GFP (Sigma-Aldrich, St. Louis, MO, USA) or IgG control overnight at 4 °C. The beads were pelleted and washed in cold lysis buffer. RNA was extracted from the immunocomplexes or from the sciatic nerves with Trizol (Thermo Fisher Scientific, Waltham, MA, USA) and reverse transcribed with SuperScript^®^ III First-Strand Synthesis System (Thermo Fisher Scientific, Waltham, MA, USA). Real-time PCR was carried out with SYBR Green I reagent (Roche USA, Indianapolis, IN, USA) on the LightCycler^®^ 480. The following primers were used for amplification: mouse β-actin (sense, GTGACGTTGACATCCGTAAA; antisense, CAGTAATCTCCTTCTGCATC) and mouse 18S (sense, GCAATTATTCCCCATGAACG; antisense, GGCCTCACTAAACCATCCAA).

## 3. Results

### 3.1. Neuron-Specific Transgene Expression Driven by AAV9.hSyn.YFP

**Figure 1:**
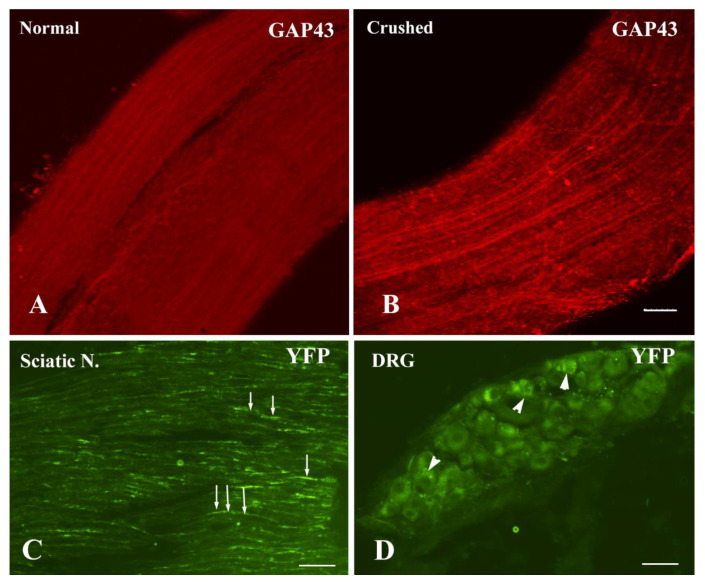
AAV9.hSyn.YFP application in the crushed sciatic nerve. (**A**) GAP 43 immunoreactivity is barely observed in the whole mount sciatic nerve of the sham control. (**B**) GAP 43+ nerve axons are readily seen in the proximal stump of the crushed nerve. (**C**) Some axons (arrows) in the proximal stump of the crushed sciatic nerve express YFP at 7 days after AAV9.hSyn.YFP injection into the crush site. (**D**) The neurons in the DRG (arrowheads) are transduced to express YFP. The punctate YFP signals are discerned in the cytoplasm. Scale bars: 100 µm.

**Figure 2:**
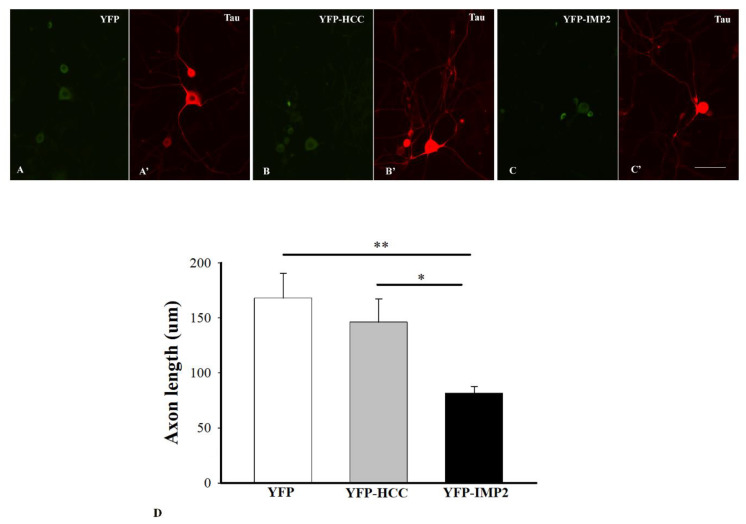
Transduction of the cultured DRG neurons by AAV9.hSyn.YFP, AAV9.hSyn.YFP-IMP2, and AAV9.hSyn.YFP-HCC. Most of the cultured DRG neurons are expressing YFP at 72 h after transduction by AAV9.hSyn.YFP. The DRG neurons transduced to express YFP (**A**), YFP-HCC (**B**), and YFP-IMP2 (**C**) are immunostained for tau (**A**’–**C**’) to delineate the cell bodies and axons. (**D**) These axons were traced and quantified with ImageJ. Quantitative analysis shows a significant decrease in average axon length of the neurons overexpressing IMP2 when compared with that in the neurons overexpressing HCC or YFP only. Mean axon length ± s.e.m. are reported (N ≥ 40, * *p* < 0.05, ** *p* < 0.01). Scale bars: 25 µm.

The sciatic nerve crush model has been widely used in the study of axon regeneration, as described [6,38,39]. GAP43 is highly expressed in regenerating axons and deemed as a regeneration marker. The whole mount sciatic nerves were immunostained for GAP43 at 7 days post injury. GAP43+ axons were readily seen in the proximal stump to the crush site, indicating they were in a regenerative process. However, GAP43 immunoreactivities were barely detectable in the sham control (Figure 1A, B). AAV serotype 9 has broad targets in the nervous system. It can transduce both neuronal and glial cells [40]. We utilized a neuron-specific promoter human synapsin in AAV9 viral vector. We examined AAV9.hSyn.YFP transduction in both the sciatic nerve crush model and injury-conditioned DRG culture. At 7 days after the nerve crush and viral injection, YFP signals were extensively visible not only in the sciatic nerve axons (Figure 1C) but also in the cell bodies of the DRG neurons (Figure 1D). However, YFP signals were only occasionally detected if the viral vector was injected into the non-crushed nerve. In the injury-conditioned DRG culture, most DRG neurons survived after AAV9 transduction. About 80% of the DRG neurons were transduced to express YFP at 4 days after the viral vector application. Although non-neuronal cells were occasionally found in the culture, none of these cells expressed the transgene. The transgene expression was exclusively in the DRG neurons and their processes (Figure 2A–C) marked by tau immunoreactivity (Figure 2A’–C’), indicating its neuron-specificity.

### 3.2. Different Effects of Overexpression of IMP2 and HCC on Axon Outgrowth

In the injury-conditioned DRG culture, the neurons extended long processes by 1 DIV. Such processes extending from the DRG neurons in vitro signify the regeneration of axons that these neurons bore in vivo [21]. Taking advantage of the neuron-specific expression of AAV9.hSyn.YFP, we applied AAV9.hSyn.YFP, AAV9.hSyn.YFP-IMP2, and AAV9.hSyn.YFP-HCC in the conditioned culture at 1 DIV and harvested the cells at 4 DIV. The entire length of axons of YFP+ neurons was well delineated by tau immunostaining. They were traced and measured with ImageJ software. Average axon length and number of axons extending from each neuron were quantified and compared among the groups treated with AAV9.hSyn.YFP, AAV9.hSyn.YFP-IMP2, and AAV9.hSyn.YFP-HCC. The adult DRG neurons overexpressing IMP2 showed a significant decrease in average axon length compared with the ones overexpressing HCC (N ≥ 40 over 5 separate cultures, * *p* < 0.05) or the ones overexpressing YFP only (** *p* < 0.01). Although the DRG neurons transduced with HCC showed a slight decrease in axon length, it was not significantly different from the neurons transduced with YFP only. There was no significant difference in neuron morphology, branching pattern, or number of axons extending from each neuron among all the groups (Figure 2D).

### 3.3. Association of Diverse Functions of IMPs in Axon Regeneration with their mRNA Binding Preferences

**Figure 3:**
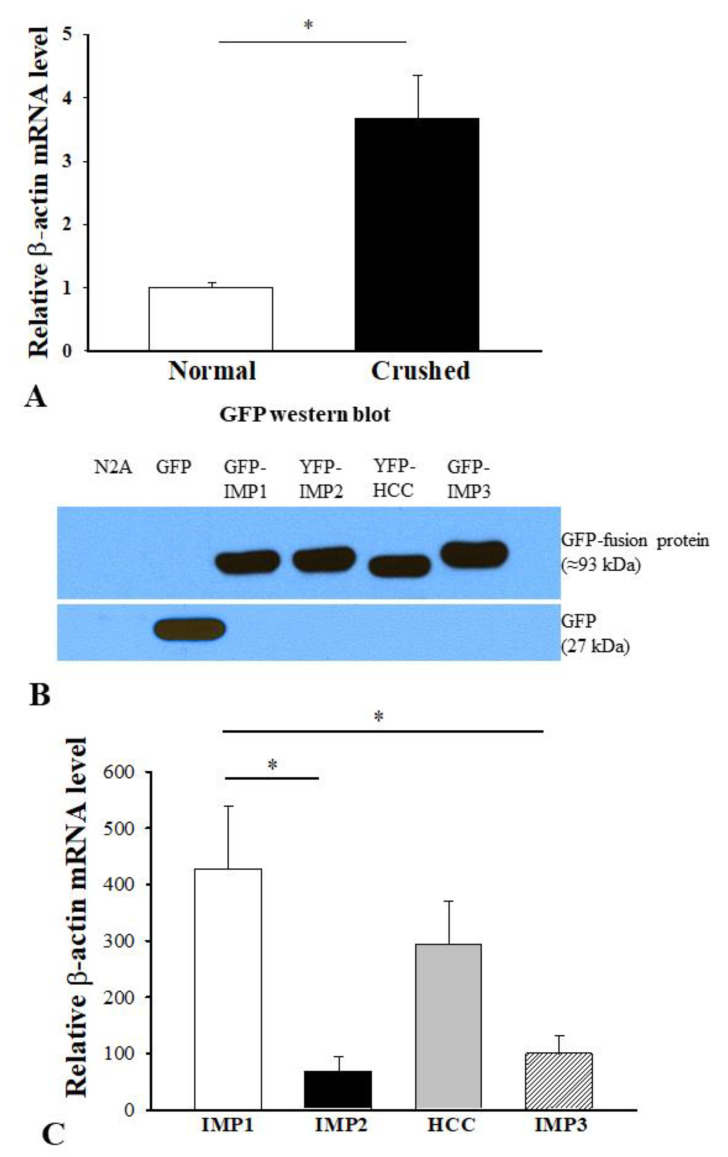
Relative quantification of β-actin mRNA in the crushed sciatic nerve and GFP immunoprecipitants from the N2A cells transfected with GFP-IMPs. (**A**) Comparative Ct method was used for relative quantification of β-actin mRNA. Level of β-actin mRNA in the crushed sciatic nerve is significantly higher than the sham control at 7 days post-surgery. The data are expressed as fold change mean ± s.e.m. (N = 5, * *p* < 0.05). (**B**) GFP Western blot confirmed transgene expression of GFP, GFP-IMP1, YFP-IMP2, YFP-HCC, and GFP-IMP3 in N2A cells. (**C**) The cell lysates were processed for immunoprecipitation with anti-GFP antibody followed by real-time PCR for β-actin mRNA. Statistical analyses of relative quantification of β-actin mRNA by one-way ANOVA with Tukey’s HSD post hoc test revealed that its levels in YFP-IMP2 and GFP-IMP3 pellets are significantly lower than that in GFP-IMP1 pellet. No statistical difference is reached when YFP-HCC is compared with YFP-IMP2 or GFP-IMP3. The data are reported as fold change mean ± s.e.m. (N = 3, * *p* < 0.05).

To explore the underlying mechanism of the inhibitory role of IMP2 during axon regeneration, we first investigated whether β-actin mRNA might be altered in response to injury. We quantified β-actin mRNA level in the sciatic nerves by real-time PCR. We used the comparative Ct method by normalizing it to 18S and compared β-actin mRNA level in the crushed with that in the sham control. The analysis showed that β-actin mRNA level was significantly increased in the crushed nerve at 5 days post-injury (N = 5, * *p* < 0.05) (Figure 3A). IMPs share a significant structural homology by having two N-terminal RNA recognition motifs and four C-terminal KH RNA binding domains, suggesting that they might target similar mRNAs. IMP1/ZBP1 is known to bind specifically to β-actin mRNA via the zipcode in the 3′UTR [41,42]. To determine if isoforms of IMPs might have similar binding affinity to β-actin mRNA, we transfected N2A cells with YFP-IMPs. Western blot analysis confirmed successful transfection and transgene expression of GFP, GFP-IMP1, YFP-IMP2, YFP-HCC, and GFP-IMP3 in N2A cells (Figure 3B). These cell lysates were further immunoprecipitated with monoclonal anti-GFP antibody followed by RNA extraction and real-time PCR for β-actin mRNA. Relative quantification of β-actin mRNA in IMP1–3 bead pellets was performed by normalizing them to N2A only and then compared with that of the GFP bead pellet. Statistical analysis revealed that β-actin mRNA level in the YFP-IMP2 pellet was significantly lower than that in the GFP-IMP1 pellet (N = 3, * *p* < 0.05). Surprisingly, the GFP-IMP3 pellet also had a significantly lower level of β-actin mRNA than the GFP-IMP1 pellet (* *p* < 0.05). Although β-actin mRNA level in YFP-HCC exhibited a higher trend, it was not of statistical difference when compared with YFP-IMP2 or GFP-IMP3 (Figure 3C).

## 4. Discussion

The neuron-specific transgene expression of IMP2 was achieved by AAV9.hSyn.YFP viral vector in this study. We examined it in both the nerve crush model and injury-conditioned DRG culture. AAV has been widely used for in vivo gene delivery. AAV9, in particular, can target the neural tissues; therefore, it provides tremendous opportunity for potential therapeutic approaches to treat neurodegenerative diseases [43]. Since AAV9 transduces both the neuronal and glial cells, utilizing neuron-specific synapsin promoter in the viral vector is necessary. Synapsin promoter driven AAV9 viral vectors have been applied by intravenous, intracerebroventricular, and spinal cord injections [32,44,45]. In this study, the viral vectors AAV9.hSyn.YFP-IMPs were inoculated in the injury-conditioned DRG culture. YFP-fused IMPs were expressed exclusively in the neuron cell bodies and axons. When AAV9.hSyn.YFP was injected into the non-crushed sciatic nerve, YFP signal was only occasionally detected in the DRG neurons or nerve axons. This is probably because the injection is a minor injury that does not disrupt the axolemma extensively enough to allow large amounts of viral vectors to penetrate for expression. However, injecting AAV9.hSyn.YFP into the crushed sciatic nerve resulted in numerous DRG neurons and their axons expressing the transgene. The result indicates that robust retrograde transport occurs after the injury and that AAV9.hSyn.YFP can drive the neuron-specific transgene expression in vivo as well. The injury-conditioned DRG culture has been widely used as an in vitro model for axon regeneration. The crush injury acutely disintegrates and destabilizes the axons and initiates vigorous anterograde and retrograde transport. It activates a regenerating process that involves a complex of cascades of molecular and cellular events [46,47]. This pre-condition sets up a regenerating process and allows us to study in the in vitro setting. The processes extending from the cultured DRG neurons are considered regenerating axons, which are immunoreactive to tau. To study a role of IMP2 in regenerating axons, the synapsin promoter-driven AAV9 was used for neuron-specific overexpression of IMP2 in the injury-conditioned DRG culture. Our result shows that IMP2 inhibits axon outgrowth from the DRG neurons, suggesting an inhibitory role in axon regeneration. However, overexpression of HCC in the injury-conditioned DRG neurons does not interrupt axon regeneration.

To investigate the potential underlying mechanism, we first examined β-actin mRNA level in response to the crush injury. The quantitative real-time PCR reveals a significant increase in β-actin mRNA in the crushed nerve compared with that in the control. This is in line with a regenerating process in the crushed nerve, which is confirmed by GAP43 upregulation. Although the increased β-actin mRNA could derive from Schwann cells or other supporting cells migrating to the site in response to the injury, contribution from the injury activated axonal transport of β-actin mRNA is unneglectable, as robust axonal mRNAs and locally translated proteins have been previously identified in the injury-conditioned DRG neurons [20]. The localized β-actin mRNA has been reported to play an important role in growth cone dynamics and axon regeneration. [6,41,42]. The zipcode region in the 3′ UTR of β-actin mRNA is a critical *cis*-element that can bind to mRNA-binding proteins, such as IMP1/ZBP1, in order to be transported in axons. In the dominant negative Tα1-GFP-3′β-actin mice, excessive 3′ UTR of β-actin mRNA competing with endogenous mRNA for IMP1/ZBP1 results in depletion of axonal β-actin mRNA and attenuated axon outgrowth of the DRG neurons, and such transport and growth deficits can be reversed by exogenous IMP1/ZBP1 [6].

In the present study, quantitative analysis of binding affinities of IMPs to β-actin mRNA shows significantly different binding affinity: IMP2 low and IMP1 high. This is consistent with a recent study that provides direct evidence showing lower affinity of KH3 and KH4 RNA binding domains of IMP2 to β-actin mRNA than those of IMP1/ZBP1 [48]. Additionally, HCC exhibits a relatively higher affinity, even though it does not reach statistical difference, most likely due to small sample size limitation. HCC, an alternative splice product of IMP2, is not endogenously expressed in normal tissues. It lacks 43 amino acids between KH2 and KH3 RNA binding domains, including a phosphorylation site at Tyr396 due to missing exon 10. Previous work has pointed out that the Tyr396 phosphorylation site is conserved among IMPs [49]. This site is important for binding to β-actin mRNA and repressing its translation. Upon phosphorylation by Src kinase, IMP1/ZBP1 releases β-actin mRNA from the complex and allows it to bind to ribosomal subunits for local translation. Non-phosphorylatable mutant IMP1/ZBP1 by converting Tyr to Ala, however, increases its affinity to β-actin mRNA and prevents its translation [42,50,51]. Additionally, RNA binding affinity and specificity also depend on the variable loops of KH domains [48]. It would not be surprising if HCC has a relatively higher affinity to β-actin mRNA than IMP2. Surprisingly, IMP3 shows a significantly lower affinity compared with IMP1/ZBP1, although it shares higher sequence homology with IMP1/ZBP1 than IMP2 or HCC. Moreover, IMP1/ZBP1 and IMP3 have almost identical developmental expression patterns, and both are reported to be potential oncogenic proteins involved in a range of cancers [5,48]. It would be worth exploring the isoform specific mechanism of regulating mRNA transport and local translation.

IMPs play differential roles during axon regeneration. Our previous result has shown that IMP1/ZBP1 can facilitate axon regeneration by rescuing the reduced axonal ß-actin mRNA localization in its haploinsufficiency [6]. Taken together with the current results, the diverse functions of IMPs seem to be correlated with their binding affinities to β-actin mRNA. In fact, β-actin mRNA is co-transported with other mRNAs in the mRNA cargos. Those mRNAs can bind to RNA binding proteins in a fashion of specific sequence preference [49]. For example, axonal GAP43 mRNA travels in the same cargos with β-actin mRNA in the IMP1/ZBP1 protein complex [6]. While an overlap exists, IMP1/ZBP1 and IMP2 respective mRNA targets have been investigated [48]. The result of this study implies that regeneration inhibitory mRNAs might dominate the cargos in the IMP2 protein complex while β-actin mRNA has low binding affinity. Additionally, RNA–protein complexes require molecular motors for transport, such as dynein, kinesins, myosins, and KIFs. These molecular motors either travel along microtubules or associate with cytoskeletal architecture [52,53]. The crush injury abruptly disrupts the subcellular organization (e.g., microtubules and neurofilaments) in the axons and drastically alters the dynamics and availability of molecular motors. Overexpressing IMP2 could compete with other mRNA-binding proteins for the molecular motors and eventually localize more regeneration inhibitory mRNAs for local translation in the injured axons.

## Data Availability

Not applicable.
